# Insights into early acne pathogenesis: Exploring intercellular dynamics and key dysregulated genes

**DOI:** 10.46439/signaling.3.053

**Published:** 2025

**Authors:** Min Deng, Kiana Farahani, George W. Agak

**Affiliations:** 1 Division of Dermatology, David Geffen School of Medicine, University of California (UCLA), Los Angeles, CA 90095, USA

**Keywords:** Acne vulgaris, Cell-cell interaction, Early-stage of acne, Cell markers in the skin, Inflammatory response, TREM2 macrophages, GRN-SORT1 axis, Hyperkeratinization, Keratinocyte, IL-13-IL13-RA1 axis

## Abstract

The comprehensive changes and shared dysregulated signaling pathways in early stage acne remains largely unexplored. In our recently published paper entitled “*Analysis of Intracellular Communication Reveals Consistent Gene Changes Associated with Early-Stage Acne Skin*,” we utilized single-cell RNA sequencing and spatial transcriptomics datasets from acne patients to analyze cell communication. We identified dysregulated genes linked to inflammatory responses and hyperkeratinization. This commentary discusses potential new markers across major skin cell types, including endothelial cells, fibroblasts, lymphocytes, myeloid cells, keratinocytes, and smooth muscle cells. Additionally, we discuss key dysregulated genes in acne lesions, focusing on the intricate interplay between inflammation and hyperkeratinization. Based on our findings, we explore potential FDA-approved treatments targeting two key pathways involved in acne pathogenesis. These insights provide new therapeutic targets for acne treatment.

## Commentary

Acne vulgaris, the most common skin condition worldwide, affects over 85% of adolescents, with nearly half continuing to experience it into adulthood. The scarring and post-inflammatory hyperpigmentation associated with acne can profoundly impact mental health and self-esteem, underscoring the importance of early and effective treatment [1].

Acne is more than a cosmetic issue; like other chronic inflammatory diseases, it involves complex interactions between host cells, dysregulated signaling pathways, genetic factors, and the microbiota [2–8]. These dynamic interactions contribute to four key pathological processes in acne-affected skin: inflammation, hyperkeratinization, seborrhea, and the accumulation of *Cutibacterium acnes* (*C. acnes*) within the pilosebaceous unit (PSU) [9]. Among them, hyperkeratinization leads to clogged pores, while seborrhea, marked by excessive sebum production, creates an environment conducive to *C. acnes* overgrowth. This bacterial proliferation and the eventual rupture of the PSU trigger an immune response, amplifying localized inflammation and further disrupting skin homeostasis. These interconnected processes underscore the complexity of acne as a disease that extends beyond its visible effects on the skin surface [10].

Building on our previous work [11], this commentary presents new findings on potential markers across major skin cell types, key dysregulated genes linked to inflammation and hyperkeratinization, and the interplay between these processes. These insights enhance our understanding of acne pathogenesis and may guide further research into inflammatory skin diseases.

## New Potential Markers for Annotating Major Skin Cell Types in Transcriptomic Data

New cell markers are essential for defining and annotating cell types in advanced technologies such as single-cell RNA sequencing (scRNA-seq) and spatial transcriptomic sequencing. These tools provide unprecedented resolution, revealing cellular heterogeneity and, enabling detailed analysis of diversity and dynamic changes that are crucial for understanding the complex process of tissue development. However, accurate cell type annotation remains challenging, as it depends on reliable markers to differentiate closely related cell types and subtypes. The lack of precise markers can lead to misclassification and obscure critical biological insights.

**Figure 1.**
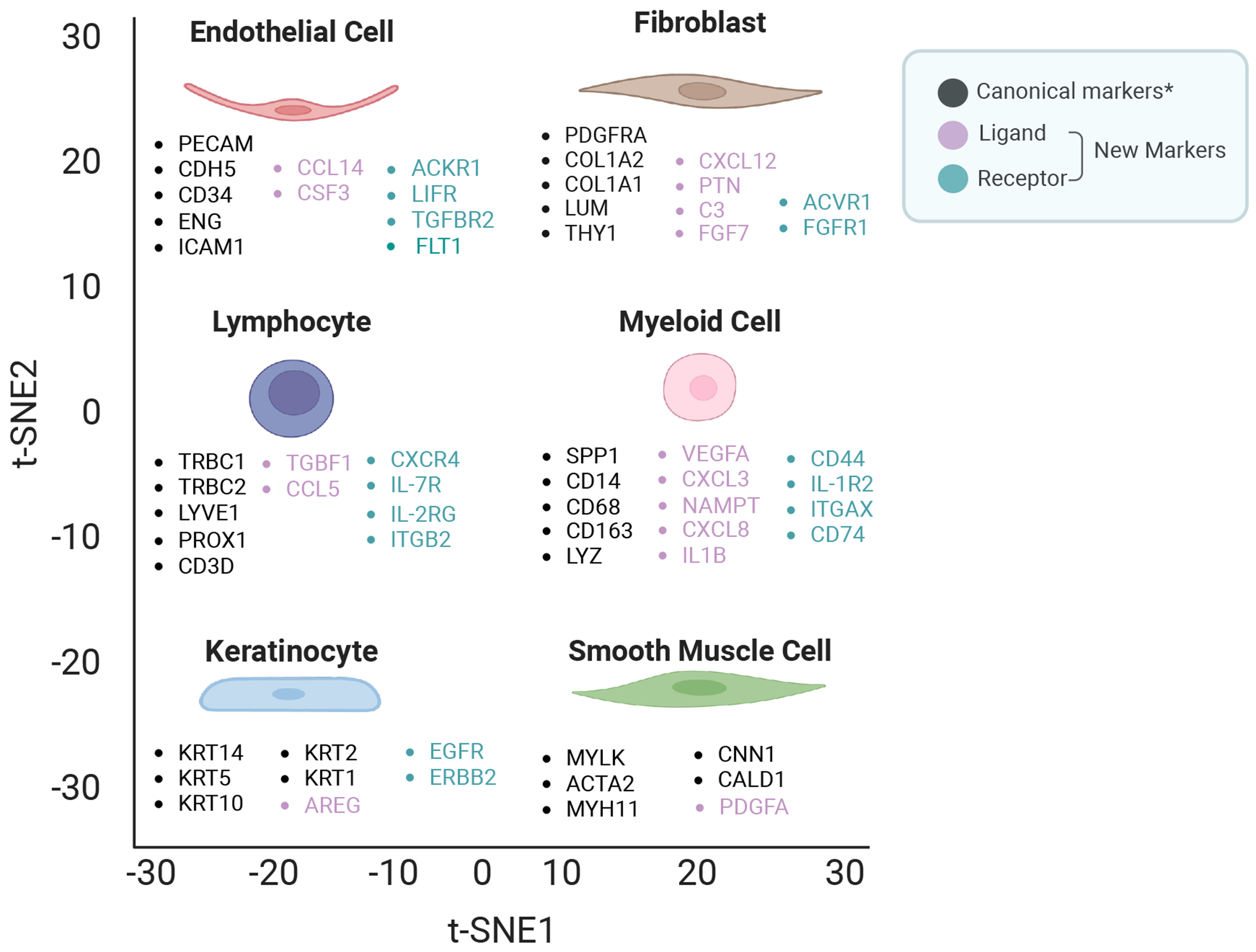
New potential markers for annotating major skin cell types in transcriptomic data. The chosen five canonical markers (black), ligands (light purple), and receptors (blue-green) are associated with various cell types including endothelial cells, fibroblasts, lymphocytes, myeloid cells, keratinocytes, and smooth muscle cells. Ligands and receptors were validated across two published skin datasets [22,62] *For simplicity, only five key canonical markers are included, though we acknowledge that additional markers have been reported in the literature. Due to page limitations, we did not subset these major cell types or list their markers.

In our study, we addressed this challenge by identifying new gene products that function as ligands and receptors in signaling pathways, offering potential cell markers for annotating major skin cell types [11]. For endothelial cells, we identified the ligands CCL14 and CSF3, along with the receptors ACKR1, LIFR, FLT1, and TGFBR2. In fibroblasts, we found ligands such as CXCL12, PTN, C3, and FGF7, as well as receptors including PDGFRA, SDC2, ACVR1, and FGFR1. Similarly, in lymphocytes, we identified the ligands TGFB1 and CCL5, and the receptors CXCR4, IL-7R, IL-2RG, and ITGB2. For myeloid cells, the ligands NAMPT, CXCL8, IL1B, VEGFA, and CXCL3 were identified alongside receptors CD74, CD44, IL-1R2, and ITGAX. In keratinocytes, we found the ligand AREG and the receptors EGFR and ERBB2, while PDGFA was identified as the ligand in smooth muscle [11] (Figure 1). These findings reveal critical roles for these genes in signal transduction and cell-specific functions, advancing our ability to annotate and understand the diverse cell types in the skin. To validate these findings, we analyzed two published skin scRNA-seq datasets and plan to use experimental approaches such as multiple channel immunofluorescence [12,13] and flow cytometry [14,15] to further validate these markers with specific cell types in skin biopsies in a future study. We will use samples from *C. acnes* and squalene-induced acne mouse models [16], with non-induced mice as controls. Additionally, biopsies from acne patients and healthy controls (individuals without acne) will be analyzed. However, obtaining human samples presents significant challenges due to ethical concerns about scarring, as patients may be reluctant to undergo skin biopsy procedures. Moreover, using the mouse model presents limitations, as acne is a uniquely human disease, and the mouse model cannot fully replicate the complex human pathogenesis of acne [16].

We also included five canonical markers for each cell type. For instance, *LUM* serves as a marker for fibroblasts in both skin and intestine, while KRT14 is included in our marker set for annotating keratinocytes in the heatmap [11,17]. Consistent with our data, *KRT14* is a well-established keratinocyte marker and is widely used in transgenic mouse models for epidermal gene knockout studies, such as those investigating Paget’s disease [18]. Integrating our novel markers with these previously identified canonical markers [19–22] will enhance the accuracy of downstream analyses and establish a robust framework for transcriptomic data (Figure 1).

## Key Genes Driving Inflammatory Responses in Acne

The inflammatory response is a key component of acne pathogenesis, driven by the interaction of *C. acnes* with host immune pathways. Toll-like receptors 2 (TLR2), predominantly expressed in macrophages surrounding the PSU [9], is a key mediator in this process. Activation of TLR2 by *C. acnes* triggers the nuclear factor kappa-light-chain-enhancer of activated B cells (NF-κB) signaling pathway. The NF-κB pathway is a central regulator of inflammation and immunity. Upon activation, it triggers a cascade of events, including the phosphorylation and degradation of IκB proteins (inhibitors of NF-κB), which sequester NF-κB in the cytoplasm. Once released, the NF-κB protein complex, comprising subunits such as p65 and p50, translocate to the nucleus. This complex acts as a transcription factor, binding to specific DNA sequences to regulate the expression of genes involved in immune responses and inflammation. In the context of acne, activation of the NF-κB pathway by TLR2 signaling leads to the production of pro-inflammatory cytokines, including IL-12, TNF-α, and IL-8 [23,24]. These cytokines contribute to the recruitment and activation of immune cells, amplifying the localized inflammatory response observed in acne lesions.

Recent advances have identified key genes that regulate inflammation in immune cells, keratinocytes, and sebocytes, significantly enhancing our understanding of acne pathophysiology. In acne, distinct cytokine networks and cellular functions are orchestrated by different cell types [25,26]. *C. acnes* strains activate immune cells to secrete innate cytokines which play critical roles in acne. *C. acnes* activate the NOD-like receptor protein 3 (NLRP3) inflammasome in monocytes, leading to the release of IL-1β and and IL-18 [27]. Conversely, IL-10, an anti-inflammatory cytokine, is downregulated in acne patients [28]. The reduction of IL-10 impairs its ability to inhibit macrophage and dendritic cell functions, including antigen presentation and the production of inflammatory mediators such as IL-12, and reactive oxygen species [27,28]. Additionally, *C. acnes* induces both Th1 and Th17 responses as evidenced by elevated IFN-γ and IL-17 in inflammatory acne lesions [29,30]. Our prior studies demonstrated that *C. acnes* ribotypes differentially regulate Th17 responses, with acne-associated strains inducing higher IL-17 levels compared to healthy [29,31]. Furthermore, *C. acnes* strains can induce Th17 cells to release antimicrobial extracellular traps which kill bacteria [32].

**Figure 2.**
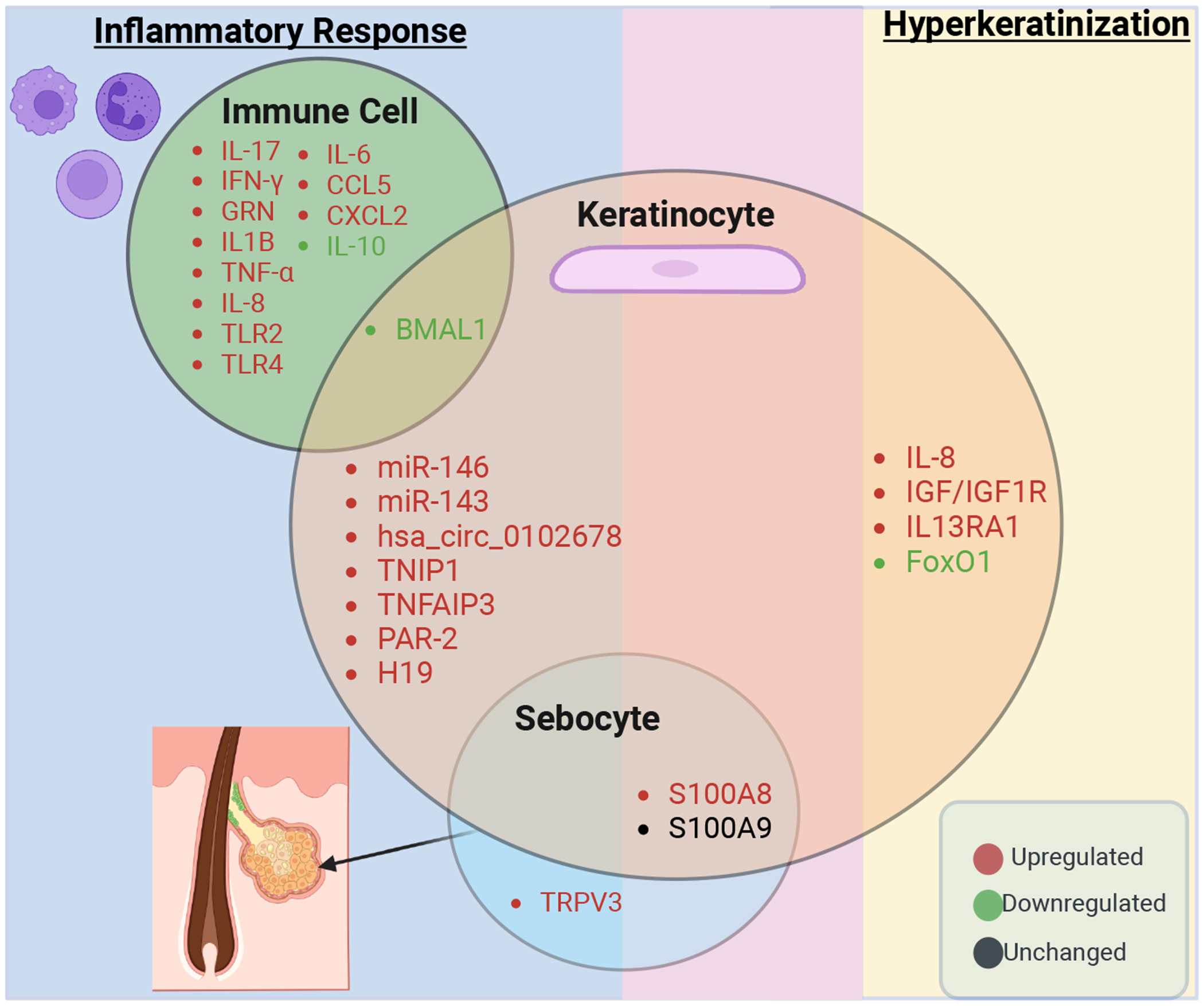
Overview of key genes involved in inflammation and hyperkeratinization in acne. The diagram lists genes from immune cells, keratinocytes, and sebocytes participating in these processes. Each circle represents a cell type, with overlapping regions indicating shared gene expression across cell types in acne. The middle overlapping light purple area between inflammation (blue) and hyperkeratinization (yellow) demonstrates that genes from sebocytes and keratinocytes contribute to both inflammation and hyperkeratinization. Genes are color-coded: Upregulated (red), downregulated (green), and unchanged (black).

Interestingly, circadian regulators such as BMAL1 have also been implicated in acne-related inflammation. BMAL1 modulates inflammatory responses in macrophages and keratinocytes, potentially influencing the expression of inflammatory cytokines via the NF-κB/NLRP3 axis [25]. The NLRP3 pathway plays a pivotal role in innate immunity by detecting cellular stress and harmful stimuli. Activation of NLRP3 triggers the assembly of the NLRP3 inflammasome, a multiprotein complex that promotes the cleavage and release of inflammatory cytokines such as IL-1β and IL-18. Beyond inflammasome activation, the NLRP3 pathway is closely linked to upstream signaling pathways, including NF-κB, which primes NLRP3 by increasing the expression of its components. Collectively, these findings highlight the complex network of genes and signaling pathways, including NF-κB, NLRP3, and associated cytokines that drive the inflammatory processes in acne pathogenesis [25] (Table 1 and Figure 2).

In keratinocytes, several genes and non-coding RNAs contribute to the inflammatory response in acne. MicroRNAs such as miR-146a and miR-143 regulate TLR2 expression and activate the IRAK1/TRAF6/NF-κB and MAPK [33,34]. Interleukin-1 receptor-associated kinase 1 (IRAK1) and TNF receptor-associated factor 6 (TRAF6) are key proteins in the TLR2 signaling cascade. Upon TLR2 activation by microbial components such as *C. acnes*, IRAK1 is recruited to the receptor complex and phosphorylated. This activates TRAF6, a ubiquitin ligase, which triggers a cascade of downstream signaling events that ultimately activate the NF-κB pathway. The MAPK pathway is another critical signal activated by IRAK1 and TRAF6 signaling. This pathway involves three primary kinase families: ERK (extracellular signal-regulated kinase), JNK (c-Jun N-terminal kinase), and p38. In acne, these kinases phosphorylate and activate transcription factors that enhance the expression of genes involved in inflammation, further amplifying the inflammatory response [33,34]. MiR-143 decreases the stability of TLR2 mRNA, reducing TLR2 protein levels and modulating inflammation [33,34]. Furthermore, the circular RNA hsa_circ_0102678 influences the miR-146a/TRAF6/IRAK1 axis to enhance inflammatory responses in keratinocytes [35]. Additional regulators such as TNIP1 upregulate multiple inflammatory pathways, including NF-κB, p38, MAPK, and JNK, contributing to keratinocyte-driven inflammation [36]. Similarly, TNFAIP3 plays a dual regulatory role by modulating both the JNK and NF-κB signaling pathways, leading to altered levels of cytokines and chemokines such as IL-6, CCL5, and IL-8 [37]. The long non-coding RNA H19, when knocked down, inhibits the miR-196a/TLR2/NF-κB axis, highlighting its pro-inflammatory role [38]. PAR-2, another key player, upregulates the IL-1β, IL-6, and TNF-α (three central pro-inflammatory cytokines) and matrix metalloproteinases (MMPs), underscoring its contribution to keratinocyte-mediated inflammation [39] (Table 1 and Figure 2).

In sebocytes, TRPV3 expression is elevated in the facial sebaceous glands of acne patients [40]. Mechanistically, TRPV3 enhances TLR2 expression by promoting the transcription factor phosphorylated FOS-like antigen-1, which binds to the TLR2 promoter, leading to TLR2 upregulation and activation of downstream NF-κB signaling pathways [40]. Stimulation of SEB-1 cells with the C. acnes strain HL043PA1 resulted in a significant upregulation of S100A8 expression, while S100A9 levels remained stable. Silencing both S100A8 and S100A9 resulted in a 50% reduction of IL-6 and IL-8 production in *C. acnes*-exposed sebocytes, indicating the proinflammatory function of S100A8/A9 in sebocytes [26]. Interestingly, the formation of extracellular traps in sebocytes following HL043PA1 treatment was observed [7], a phenomenon previously described by Agak *et al.* in immune cells such as Th17 cells [32]. This observation expands our understanding by demonstrating that some non-immune cells, such as sebocytes, can also release extracellular traps as part of their defense against *C. acnes*, suggesting a broader role for these traps in skin immunity (Table 1 and Figure 2). Additionally, S100A8 and S100A9 have been shown to stimulate keratinocyte proliferation via the MAPK pathway suggesting their involvement in hyperkeratinization [31]. These findings indicate that S100A8 and S100A9 not only contribute to inflammation but may also play a pivotal role in linking hyperkeratinization and inflammation [26,41]. This dual role underscores their potential as key mediators in acne (Tables 1, 2, and Figure 2).

In our study, we performed a differential analysis of altered signaling pathways, identifying 26 genes with significantly changed expression levels in acne lesions compared to normal skin [11]. We then focused on 10 genes predominantly expressed in myeloid cells and lymphocytes due to their strong association with inflammation. Among these, granulin precursor (*GRN*) was consistently dysregulated among all six lesional samples compared to non-lesional samples. Further analysis revealed that *GRN* and its receptor, Sortilin 1 (*SORT1*), were upregulated in TREM2-expressing macrophages [11]. Treatment with *GRN* led to increased expression of three central pro-inflammatory cytokines and chemokines, including *IL-18*, *CCL5*, and *CXCL21*. These findings suggest that the *GRN-SORT1* axis plays a critical role in amplifying the inflammatory response in *TREM2* macrophages, potentially exacerbating inflammation in acne lesions [11]. Consistent with our study, *GRN* was primarily expressed in macrophages across multiple organs, including the brain [42], pancreas [43], and liver [44], indicating its critical role in macrophage function. Further research is needed to explore *its* contribution to acne formation *in vivo*.

## The Intersection of Hyperkeratinization and Inflammation in Acne

Hyperkeratinization in acne involves an abnormal increase in keratin production within hair follicles, leading to clogged pores that set the stage for comedone formation. This process is triggered by hormonal shifts, especially during adolescence, which increase keratinocyte proliferation and disrupt normal desquamation [45]. As keratin and sebum accumulate within the follicle, they create an occlusive environment that not only obstructs the pore but also traps *C. acnes* which thrives in lipid-rich conditions [10]. In the context of acne, key signaling pathways, including the MAPK and PI3K/AKT/FoxO1 axes, are implicated in keratinocyte hyperproliferation, which can lead to hyperkeratinization [45]. For example, IGF-1 and IL-8 activate AKT signaling to regulate FoxO1 activity, while downregulation of FoxO1 results in increased keratinocyte proliferation and excessive keratin production, further promoting follicular occlusion [46–48] (Table 2 and Figure 2). Beyond their function in hyperkeratinization, keratinocytes also actively mediate immune signaling [49]. Upon exposure to *C. acnes*, keratinocytes release three central pro-inflammatory cytokines, signaling immune cells to migrate to the follicle [49]. This immune recruitment promotes inflammation, transforming a non-inflammatory comedone into a papule or pustule [10]. Additionally, keratinocytes produce antimicrobial peptides (AMPs), including LL-37 and β-defensin, which may help counteract *C. acnes* growth [50]. However, while these AMPs are protective in the skin, they also exacerbate local inflammation through stimulating cytokine/chemokine production and participate in wound healing by promoting keratinocyte migration and proliferation, creating a cycle in which the immune response perpetuates follicular hyperkeratinization and inflammation, highlighting the intricate interplay between these processes [51].

Hyperkeratinization is fundamentally driven by the dysregulation of keratinocyte signaling pathways. Recent research, including our own analysis of published scRNA-seq datasets, identified the *IL-13-IL13RA1* axis—a critical component of the type 2 immune pathway—as a key modulator of keratinocyte function in acne [11]. Activation of this pathway disrupts the expression of essential differentiation genes such as KRT16, KRT17, and FLG, leading to impaired keratinocyte differentiation and abnormal keratin accumulation [11].

The *IL-13-IL13RA1* axis may influence acne through multiple mechanisms. It regulates lipid metabolism in sebocytes [52], impairs skin barrier function by downregulating *FLG* expression [53], and contributes to tissue remodeling and fibrosis [54]. As *IL-13* is secreted by mast cells, NKT cells, T cells, and neutrophils [11], this pathway links immune signaling to hyperkeratinization. These findings suggest that immune polarization towards a type 2 response may promote keratinocyte proliferation and play a role in the development of acne lesions.

## Connecting Acne Pathogenesis to Therapeutic Strategies

Based on our findings, we discuss the potential FDA-approved treatments targeting two key pathways identified in acne pathogenesis. One promising clinical application involves dupilumab, an FDA-approved monoclonal antibody that inhibits IL-13 and IL-4 signaling by blocking the IL-4 receptor alpha subunit. While dupilumab is approved for the treatment of atopic dermatitis [55], asthma [56], and chronic rhinosinusitis [57] with nasal polyps, it holds significant potential for treating inflammatory acne. By targeting IL-13, dupilumab may reduce inflammation, restore skin barrier function, and regulate sebocyte activity, offering a novel approach to mitigating acne pathogenesis through modulation of the IL-13-IL13RA1 axis.

Currently, there are no FDA-approved treatments specifically targeting GRN or its receptor SORT1. However, targeting the downstream cytokines regulated by the GRN-SORT1 axis could present a promising therapeutic strategy. The GRN-SORT1 axis influences several pro-inflammatory mediators, including the cytokines IL-1β, TNF-α, IL-6, IL-18 and the chemokines CCL5, and CXCL2 [11]. For example, Maraviroc, an FDA-approved CCR5 antagonist for HIV, blocks the receptor for CCL5 [58] and may reduce the chemotactic effects of CCL5 in acne lesions. Similarly, TNF-α inhibitors such as Infliximab, Adalimumab, and Etanercept, widely used in the treatment of autoimmune diseases such as rheumatoid arthritis [59], can help reduce inflammation by inhibiting TNF-α signaling. IL-6 receptor antagonists, Tocilizumab and Sarilumab, approved for conditions such as rheumatoid arthritis [60], could also offer potential benefits in reducing inflammation in acne. While these therapies were developed for other inflammatory conditions, they provide a foundation for repurposing existing treatments to target the inflammatory pathways that contribute to acne. Notably, IL-1β inhibitors, such as Anakinra and Canakinumab, have shown promise in treating acne [61].

Existing FDA-approved therapies targeting the cytokines above present a potential strategy for treating acne. Future clinical research may further validate their efficacy in acne management.

## Conclusion

In summary, our findings highlight the importance of understanding cellular communication within acne lesions and identifying novel markers and dysregulated genes that differentiate healthy and acne-prone skin. We also discussed potential FDA-approved treatments targeting two key pathways involved in acne pathogenesis. These insights reveal critical signaling pathways and offer new therapeutic targets for acne treatment.

## Figures and Tables

**Table 1.** Key genes that participate in inflammation in acne.

Gene	Acne model or cell type	Expression Change	Mechanism
TLR2 [23]	Peritoneal macrophage, RAW 264.7 cell, primary human monocyte	Upregulated	Receptor for *C. acnes* and activate NF-κB signaling
IL-17 [30,31]	Th17 and Th1 cells	Upregulated	Promotion of Th17 Cell Differentiation; Induce the expression of three central pro-inflammatory cytokines
IFN-γ [63]	CD4^+^ T cells	Upregulated	Mediate Th1 cell differentiation and response; activate immune cells including natural killer cells and macrophages to present antigens, and release cytokines including IL-12 [64,65]
IL-10 [24,66]	PBMCs (CD4^+^ T cells, Tregs, monocytes)	Downregulated	Inhibit macrophage and dendritic cell functions by downregulating antigen presentation as well as the production of IL-12, chemokines, nitric oxide, reactive oxygen species and costimulatory molecules. *C. acnes* strains associated with healthy skin upregulate IL-10 expression
GRN [11]	TREM2 macrophage	Upregulated	Increase the expression of cytokines IL-18 and chemokines CCL5 and CXCL2
IL-1β [27]	Primary human monocyte	Upregulated	Activate NLRP3 inflammasome
BMAL1 [25]	C57BL/6 mice, RAW264.7 cell, Primary mouse keratinocyte	Downregulated	Inhibit NF-κB/NLRP3 axis
miR-146a [33]	Primary human keratinocyte	Upregulated	Activate the TLR2/IRAK1/TRAF6/NF-κB and MAPK pathways.
miR-143 [34]	Tlr2^−/−^ mice and NHEK cell	Upregulated	Decrease the stability of TLR2 mRNA and then decreased TLR2 protein
hsa_circ_0102678[35]	Primary human keratinocyte	Upregulated	Regulate miR-146a/TRAF6 and IRAK1 axis
TNIP1 [36]	HPV-KER and NHEK cell	Upregulated	Upregulate the NF-κB, p38, MAPK and JNK pathways
TNFAIP3 [37]	HPV-KER cell	Upregulated	Dually regulate JNK and NF-κB signaling
PAR-2 [39]	HaCaT	Upregulated	Regulate the expression of three central pro-inflammatory cytokines, hBD-2, LL-37, MMP-1, −2, −3, −9, and −13
H19 [38]	HaCaT cell	Upregulated	Regulate the miR-196a/TLR2/NF-κB Axis.
TRPV3 [40]	Acne mice model and Sebocyte	Upregulated	Lead to TLR2 upregulation and downstream NF-κB signaling activation
S100A8 [26]	Sebocytes	Upregulated	Promote the expression level of IL-8 and IL-6
S100A9 [26]	Sebocytes	Unchanged	

HPK: Human Primary Keratinocyte; NHEK: Normal Human Epidermal Keratinocytes; HPV-KER: Human Papillomavirus-Immortalized Keratinocyte; PBMCs: Peripheral Blood Mononuclear Cells; Tregs: Regulatory T cells

**Table 2.** Indicator of inflammation-driven keratinocyte activation.

Gene	Acne model or material used	Expression Change	Mechanism
S100A8 [26,41]	HaCaT cell	Upregulated	Promote inflammation and MAPK pathway
S100A9 [26,41]	HaCaT cell	Upregulated	
IL-8 [24,46]	HaCaT cell	Upregulated	Activate inflammation and AKT/FOXO1 axis
IGF-1/IGF1-R [47]	Skin biopsies and NHEK cell	Upregulated	Regulate inflammation and induce proliferation of keratinocytes through PI3K/Akt/FoxO1
FoxO1 [48,67]	HPK	Downregulated	Regulate inflammation and promote differentiation and apoptosis in HPKs
IL13RA1 [11]	HaCaT cell	Upregulated	The ligand IL-13 can be released by mast cells, NK cells, and regulate KRT17, KRT16, and FLG expression.

## Abbreviations:

C. acnes: Cutibacterium acnes
scRNA-seq: Single-cell RNA sequencing
SORT1: Sortilin 1
TLRs: Toll-like receptors
NF-κB Signaling Pathway: Nuclear Factor Kappa-Light-Chain-Enhancer of Activated B cells Signaling Pathway
NLRP3: NOD-like Receptor Protein 3
MAPK Pathway: Mitogen-Activated Protein Kinase Signaling Pathway
AMPs: Antimicrobial Peptides
HPK: Human Primary Keratinocyte
NHEK: Normal Human Epidermal Keratinocytes
HPV-KER: Human Papillomavirus-Immortalized Keratinocyte
PBMCs: Peripheral Blood Mononuclear Cells
GRN: Granulin Precursor
